# Protective effect of isoflavones and triterpenoid saponins from pueraria lobata on liver diseases: A review

**DOI:** 10.1002/fsn3.2668

**Published:** 2021-12-10

**Authors:** Heng He, Shuwei Peng, Xu Song, Renyong Jia, Yuanfeng Zou, Lixia Li, Zhongqiong Yin

**Affiliations:** ^1^ Natural Medicine Research Center College of Veterinary Medicine Sichuan Agricultural University Chengdu China; ^2^ Key Laboratory of Animal Disease and Human Health of Sichuan Province Sichuan Agricultural University Chengdu China

**Keywords:** liver diseases, natural products, pueraria lobata, puerarin

## Abstract

In recent years, with the improvement of people's living standard and the change of diet structure, liver disease and its related complications have become a significant public health problem globally. Pueraria lobata (*Pueraria montana var. lobata (Willd.) Sanjappa & Pradeep*) belongs to the genus Pueraria, which is widely planted and used as medicine and food in Asia with a long history. A variety of natural active products, including puerarin, daidzein, formononetin, genistein, and soyasaponin, have been isolated and identified from pueraria lobata. A large number of studies have shown that various natural active products of pueraria lobata can play a protective role in different types of liver diseases by regulating oxidative stress, inflammatory response, lipid metabolism, etc. In this review, we focused on the protective effects of isoflavones and triterpenoid saponins from pueraria lobata on the liver through different targeted therapeutic mechanisms. What's more, we summarized their therapeutic potential for different types of liver diseases to provide evidence for their clinical application.

## Graphical abstract

BLURB FOR ETOCThis review focuses on the protective effect of isoflavones and triterpenoid saponins from pueraria lobata on the liver and their therapeutic potential for different types of liver diseases.

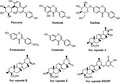

## 1 INTRODUCTION

The liver is one of the most important organs in mammals. As the central metabolic hub, it also serves as a detoxification function. Nowadays, liver diseases remain an important public health problem in the world. In addition to endangering human life and health, it also imposes a substantial medical and economic burden on the world (Asrani et al., 2018; Blachier et al., 2013; Stepanova et al., 2017; Williams et al., 2018). There are many causes of liver disease, which are mainly divided into alcoholic liver diseases (ALD), nonalcoholic fatty liver diseases (NAFLD), drug‐induced liver injury (DILI), viral hepatitis, autoimmune liver diseases, and cholestatic liver diseases (Grønbæk et al., 2014; Kim et al., 2018; Trauner et al., 2008; Williams et al., 2020). DILI (especially acetaminophen toxicity) and viral hepatitis are the leading causes of acute liver failure (ALF) (Lefkowitch, 2016). Although the harm of liver diseases has been realized all over the world, there is still a lack of comprehensive public health responses and treatment strategies, so it is urgent to seek new drugs for liver diseases treatment (Lazarus et al., 2020; Williams et al., 2020).

Pueraria lobata, commonly known as kudzu, is a legume vine native from Southeast Asia as one of the first medicinal plants used in traditional Chinese medicine (Croom, 2004; Takano et al., 2013). It has been recorded from Asia, Europe, and America, and the earliest written records of its use in China can be traced back to 1000 BC (Keung & Vallee, 1998). At present, Pueraria lobata has been included in Chinese pharmacopeia. In addition to treating diseases such as diabetes and cardiovascular diseases, Pueraria lobata has also been used to cure liver injury caused by alcohol abuse due to its function of relieving alcoholism and protecting liver (Maji et al., 2014; Wang et al., 2021; Wong et al., 2011). As well as being a therapeutic drug, Pueraria lobata is widely used in nutritious foods and dietary supplements (Dong xueqian et al., 2020; Xin Li et al., 2017).

**FIGURE 1:**
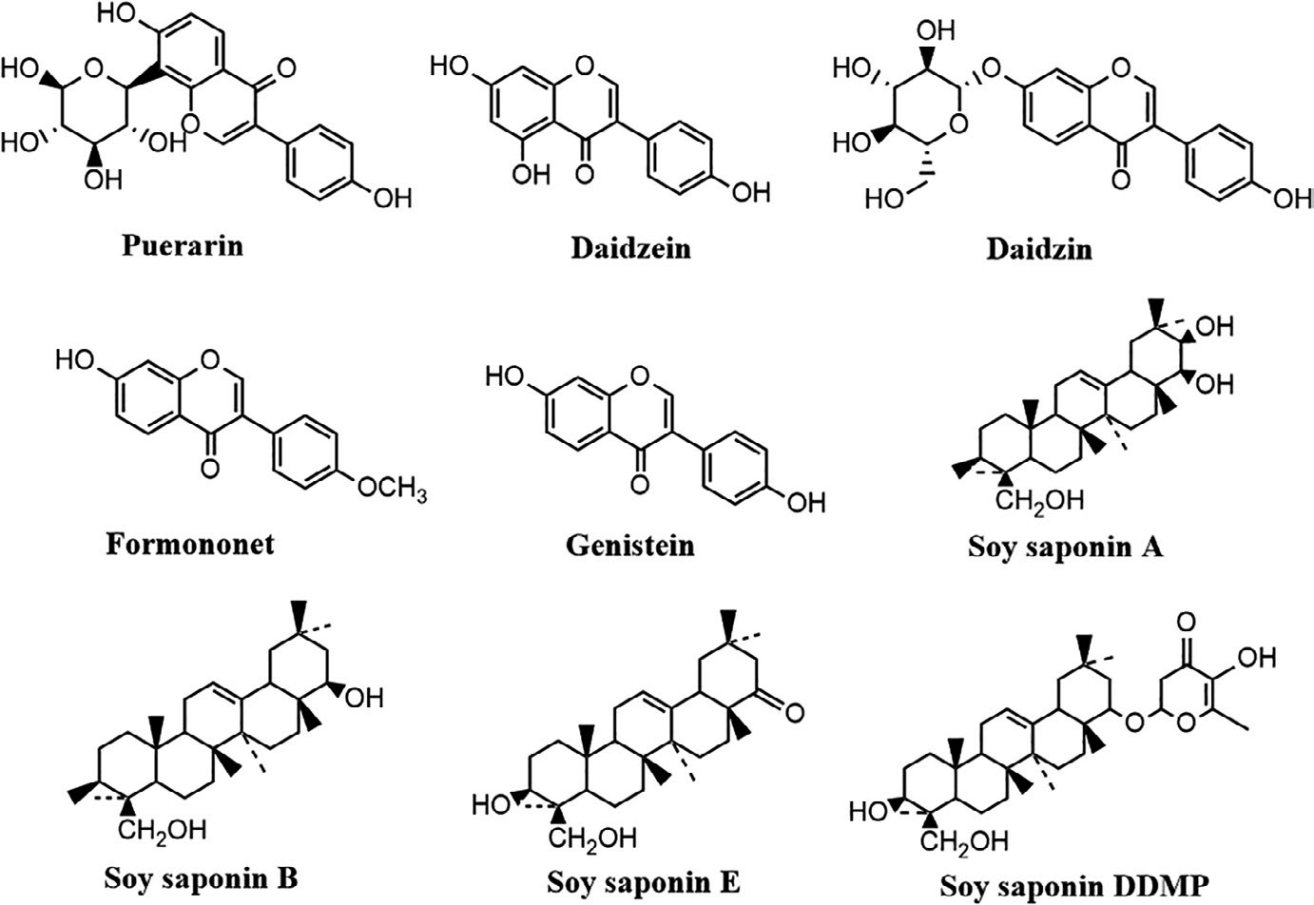
Chemical structures of the main compounds in Pueraria lobata

To elucidate the mechanism of action of traditional Chinese medicine, it is necessary to identify the active ingredients, which can not only be the scientific basis for the pharmacological action of Chinese herbs, but also provide new ideas for the research and development of new drugs. At present, more than 70 kinds of phytochemicals have been identified in Pueraria lobata, which can be divided into four groups according to their chemical constituents, namely isoflavones, triterpenes, coumarins, and alkaloids (Croom, 2004; Lina et al., 2019). Chemical structures of major natural products are shown in Figure 1. Isoflavones and triterpenoid saponins are considered as the main active components in Pueraria lobata. The isoflavones in Pueraria lobata mainly include puerarin, daidzin, daidzein, genistein, and formononetin, while triterpenoid saponins mainly include soyasaponin and pueraria saponin (Zhiping et al., 2010). This review focuses on the protective effect of isoflavones and triterpenoid saponins from Pueraria lobata on the liver and their therapeutic potential for different types of liver diseases.

### 1.1 Hepatoprotective effects of active compounds in Pueraria lobata

#### 1.1.1 Puerarin

Puerarin, the first ingredient isolated from pueraria lobata in the late 1950s, is the most abundant secondary metabolite in pueraria lobata (Zhou et al., 2014). It is widely used in the clinical treatment of cardiovascular and cerebrovascular diseases, diabetes, osteonecrosis, cancer and has a protective effect on liver injury caused by various factors (Wei et al., 2014; Zhou et al., 2014). Meanwhile, puerarin has a protective effect on liver injury caused by various factors. Puerarin has low solubility and permeability, leading to poor absorption and low bioavailability in the gastrointestinal tract. When adult female rats were given puerarin 5 and 10 mg/kg orally, the absolute oral bioavailability was about 7%, which was predominantly excreted through conversion into glucuronide metabolites, mostly excreted through the urinary system, and a little excreted through the hepatobiliary pathway (Anukunwithaya et al., 2018; Luo et al., 2010; Zhang, 2019). With considerable progress in the bioconversion of puerarin structure by microorganisms and free enzymes, more and more new derivatives of puerarin with high solubility and biological activity have been developed, which have promoted the clinical application of puerarin (Liu et al., 2016).

##### Effects of puerarin on alcoholic liver disease

ALD is one of the leading causes of chronic liver disease in the world, leading to liver fibrosis and cirrhosis. It has been reported that the pathogenesis of ALD includes oxidative stress, liver cell metabolic disorders, liver steatosis, and inflammation (Gao & Bataller, 2011; Louvet & Mathurin, 2015; Penny, 2013).

**FIGURE 2:**
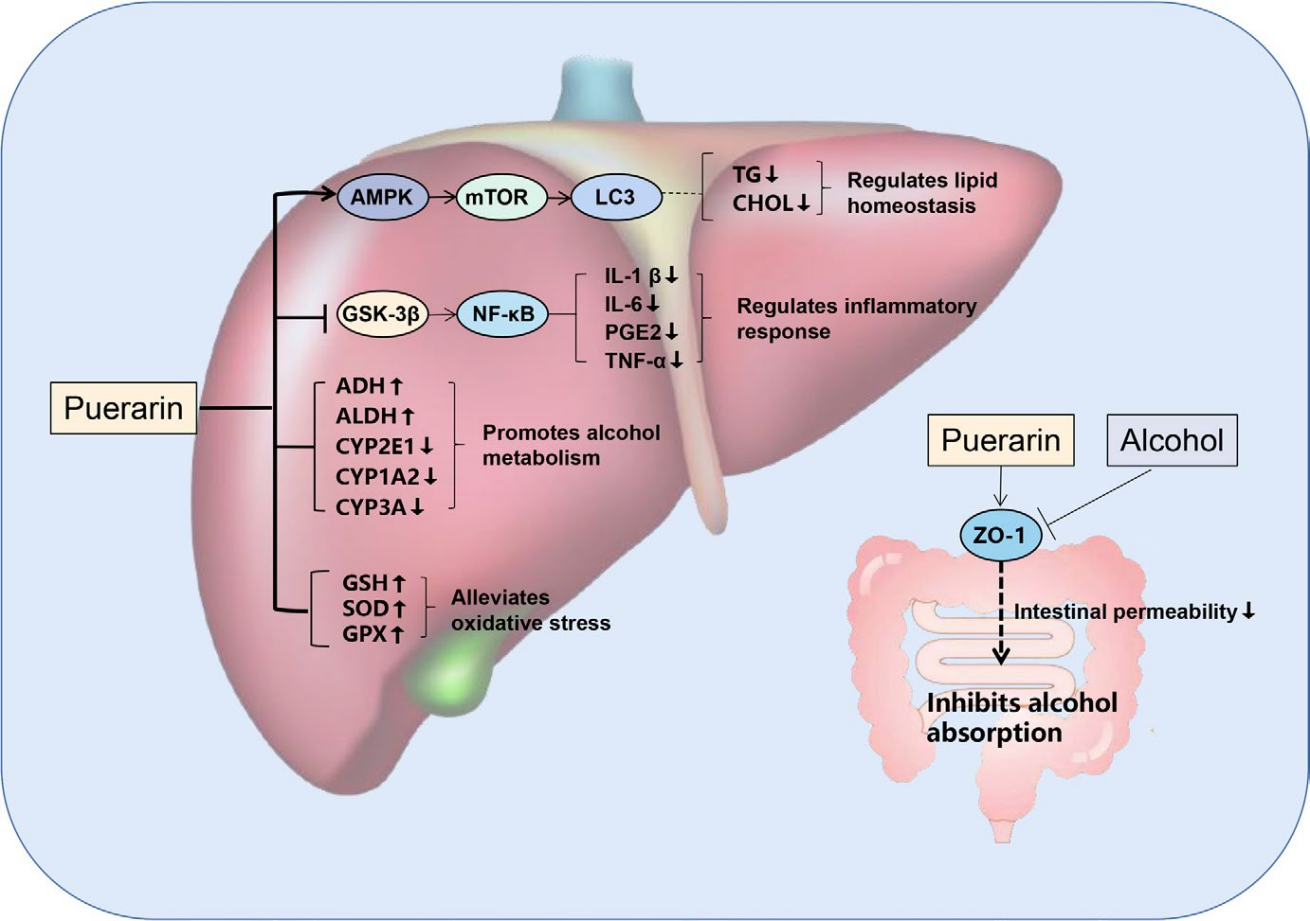
The potential protective mechanism of puerarin against ALD. AMPK, adenosine monophosphate‐activated protein kinase; mTOR, mammalian target of rapamycin; LC3, microtubule‐associated protein 1A/1B‐light chain 3; TG, triglyceride; CHOL, cholesterin; GSK‐3β, glycogen synthase kinase‐3β; NF‐κB, nuclear factor kappa‐B; IL‐1, interleukin 1; IL‐6, interleukin 6; PGE2, prostaglandin E2; TNF‐α, tumor necrosis factor‐α; SOD, superoxide dismutase; GPX, glutathione peroxidase; ZO‐1, zonula occluden 1

At present, there are many researches on the protective effect of puerarin on alcoholic liver disease, involving multiple mechanisms (Figure 2). Puerarin can delay gastric emptying, slow down the absorption rate of alcohol, reduce intestinal wall permeability, and protect gastrointestinal mucosa (Lin & Li, 1998; Zhang et al., 2009). Meanwhile, puerarin can also regulate the activity of enzymes related to ethanol metabolism, such as ethanol dehydrogenase (ADH) and aldehyde dehydrogenase (ALDH), and selectively inactivate Cytochrome P450 2E1(CYP2E1), thus reducing the production of free radicals in ethanol catabolism pathway and alleviating the damage of liver cells caused by immune stress (Chen et al., 2013). What's more, puerarin can improve the activity of antioxidant enzymes in the body and reduce oxidative stress in tissues caused by alcoholic liver injury (Zhao et al., 2010). Previous studies have found that the expression levels of alanine aminotransferase (ALT), aspartate aminotransferase (AST), alkaline phosphatase (ALP), and proinflammatory cytokine were significantly reduced in the puerarin‐treated group compared with the control group in the model of chronic alcohol‐induced liver injury (Li et al., 2013). This suggests that puerarin can reduce the liver injury caused by chronic alcohol by regulating the inflammatory response. Another study showed that puerarin could significantly restore the activity of ethanol‐treated hepatocytes, reduce the lipid accumulation in cells compared with untreated control, and recover the autophagy activity of cells by stimulating AMPK and inhibiting mTOR (Noh et al., 2011). In a pilot study, it was found that treating with puerarin can change drinking habits and reduce alcohol consumption in humans (Penetar et al., 2012). These results indicate that puerarin can be used in the treatment of alcoholic liver injury.

##### Effects of puerarin on oxidative stress in liver

**FIGURE 3:**
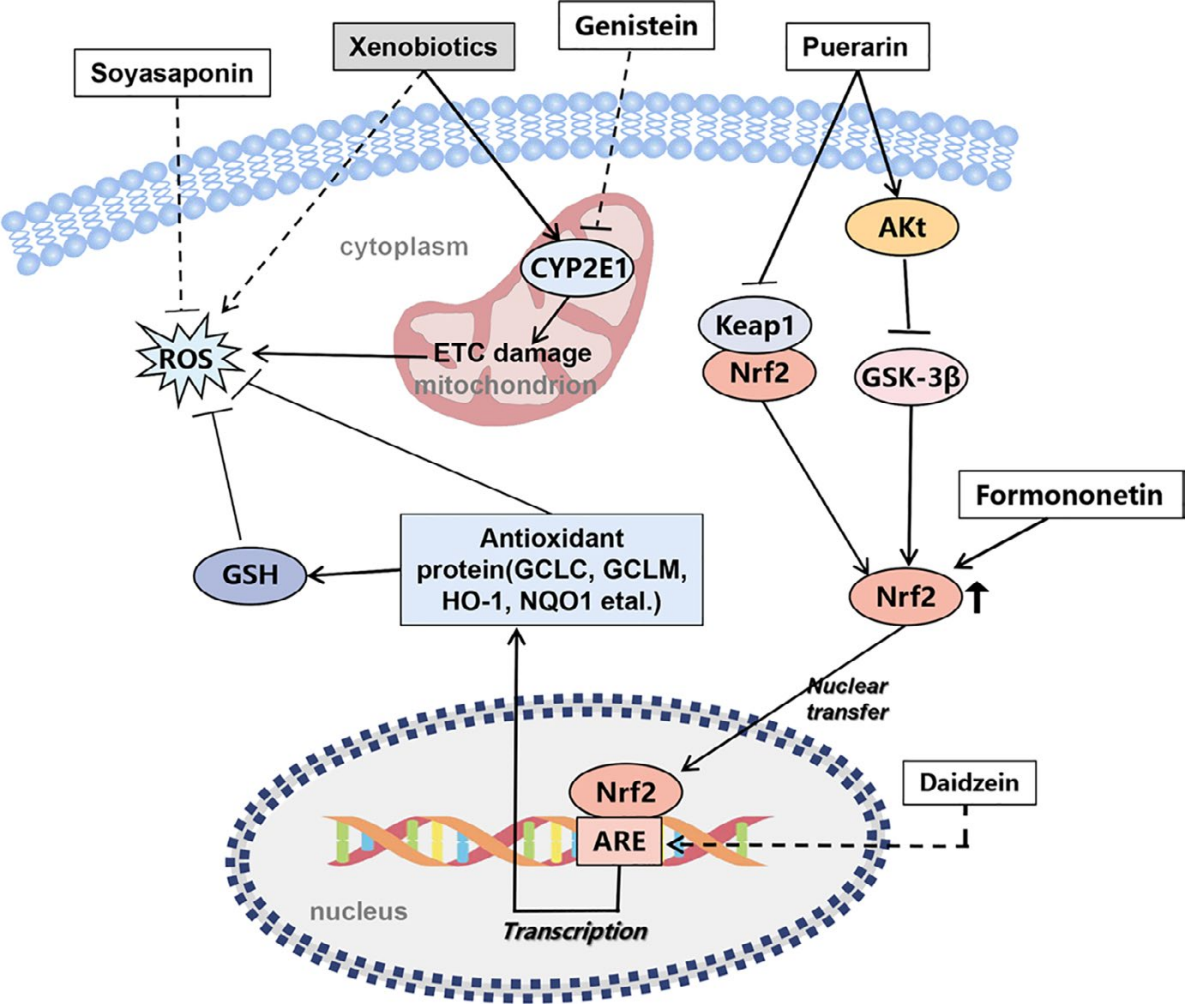
Networks of molecular signaling underlying antioxidation of active compounds in Pueraria lobata. ETC, electron transport chain; Akt, protein kinase B; GCLC, glutamate cysteine ligase catalyzes subunit; GCLM, glutamic cysteine ligase‐modulated subunit; HO‐1, hemeoxygenase‐1; NQO1, NAD(P)H quinone oxidoreductase 1; Nrf2, nuclear erythroid‐2‐related factor 2; Keap1, Kelch‐like ECH‐associated protein 1; ARE, Antioxidant responsive element

Puerarin has potential antioxidative activity both in vitro and in vivo (Figure 3). Glutathione (GSH) plays a vital role in the antioxidant system which can significantly promote the clearance of reactive oxygen species (ROS). It can also maintain the balance of the intracellular redox state and is essential for nuclear function and cell survival under oxidative stress (Hatem et al., 2014; Jozefczak et al., 2012). Glutathione/oxidized glutathione (GSH/GSSG) ratio is one of the important determinants of oxidative stress in vivo (Villaverde et al., 2004). Heavy metal ions can produce a large number of reactive radicals by consuming glutathione and protein‐bound sulfhydryl groups, which eventually lead to lipid peroxidation and DNA damage (Fu & Xi, 2020). Puerarin also has a protective activities on liver damage caused by the poisoning of various heavy metals. One study showed that puerarin could reduce part of the oxidative stress response via increasing the activity of the antioxidant enzyme, significantly increasing the GSH and GSH/GSSG ratio in the liver of lead‐treated rats, and reducing the activity of caspase‐3 to resist DNA damage and apoptosis (Liu et al., 2011, 2012). Metallothionein (MT) protects cells from the influence of oxidants and electrophiles by scavenging free radicals (Ruttkay‐Nedecky et al., 2013). Due to its high affinity to heavy metals, metallothionein plays an essential role in detoxifying heavy metals and maintaining the homeostasis of base metal ions (Birben et al., 2012). Puerarin significantly increased MT levels in the liver of mice exposed to nickel, thereby protecting liver cells from oxidative stress‐induced damage (Liu et al., 2016).

In addition, puerarin can also have a protective effect on CCl_4_‐induced liver injury via restoring GSH level and total antioxidant capacity; meanwhile, it can reduce hyperlipidemia via regulating lipid peroxidation through the JNK/c‐jun/CYP7A1 pathway (Ma, Ding, Zhao, et al., 2014). Other studies have shown that puerarin can up‐regulate B‐cell lymphoma‐2 (BCL‐2) to inhibit oxidative stress, improve liver energy metabolism dysfunction, and have a protective effect on liver oxidative damage in mice with type 2 diabetes mellitus (T2DM) (Shuo Yang et al., 2009).

Nrf2 is an important nuclear transcription factor involved in the expression of a variety of proteins, which is considered as the central regulator of oxidative stress response in the body (Kaspar et al., 2009). It has been suggested that puerarin can promote the expression of glutamate cysteine ligase catalyzes subunit (GCLC) by promoting the nuclear transport of Nrf2 in PC12 cells, thus increasing the level of GSH (Li et al., 2014). Although there is no direct evidence that puerarin can exert a protective effect on oxidative stress damage of the liver through Nrf2, some studies have already proved that it can regulate the oxidative stress response in multiple parts of the body by activating the Nrf2 signaling pathway (Jadeja et al., 2016; Ma et al., 2014; Zhang et al., 2015; Zhou et al., 2014; Zou et al., 2013). Therefore, we speculate that Nrf2 may be a new target for preventing and treating liver oxidative stress.

##### Effects of puerarin on hepatic lipid metabolism

**FIGURE 4:**
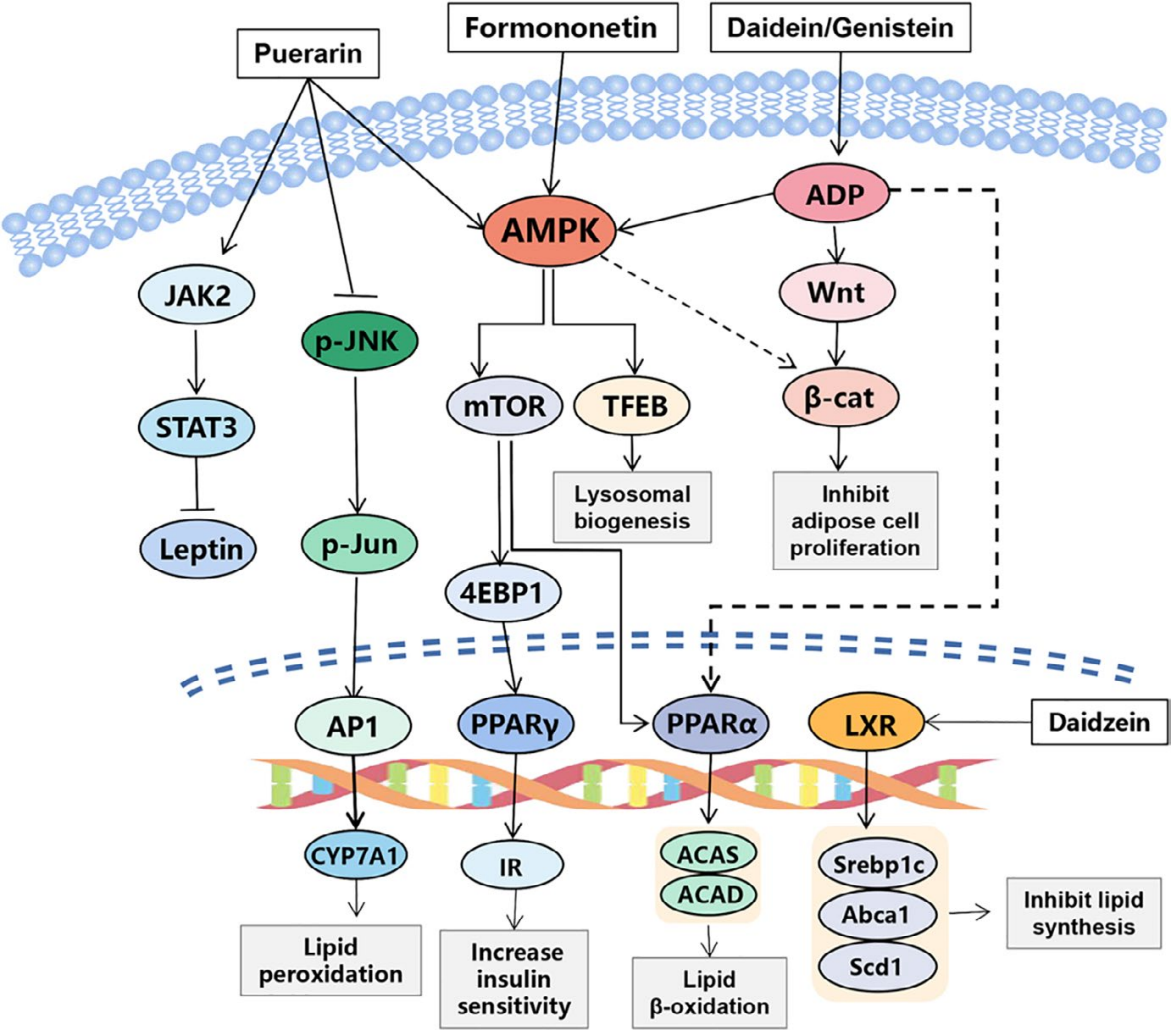
Networks of molecular signaling underlying regulative effects of active compounds in Pueraria lobata on hepatic lipid metabolism. JAK2, janus activating kinase 2; STAT3, signal transducer and activator of transcription 3; JNK, c‐Jun N‐terminal kinase; AP1, activator protein 1; IR, insulin resistance; PPARγ, peroxisome proliferators‐activated receptors‐γ; TFEB, transcription factor EB; 4EBP1, eukaryotic translation initiation factor 4E‐binding protein 1; ADP, adenosine diphosphate; β‐cat, β‐catenin; Srebp1c, sterol regulatory element‐binding protein‐1c; Abca1, ATP‐binding cassette transporter A1; Scd1, stearoyl‐CoA desaturase 1

Unhealthy eating habit is an important cause of NAFLD, which is often accompanied by obesity and diabetes (Dowman et al., 2010; Tiniakos et al., 2010). The potential mechanisms of puerarin on liver lipid metabolism are shown in Figure 4. In mice which feed with a continuous high‐fat diet, it can inhibit fatty acid synthetase activity, activate the AMPK, carnitine acyltransferase (CAT) and hormone‐sensitive lipase (HSL), and reduce liver lipid accumulation and steatosis by improving leptin signaling in the JAK2/STAT3 pathway treating with puerarin (Zheng et al., 2015; Zheng et al., 2009). Other studies have shown that puerarin has beneficial effects on NAFLD by increasing the expression levels of liver peroxisome proliferator‐activated receptor (PPAR) and insulin receptor (IR) (Zhao, Liu, et al., 2016). In addition, puerarin also regulated lipid metabolism via the PPAR pathway in hepatitis mice induced by a methionine–choline‐deficient diet, suggesting that puerarin may help prevent liver disease caused by dietary habits (Wang et al., 2014).

##### Effects of puerarin on hepatic fibrosis

**FIGURE 5:**
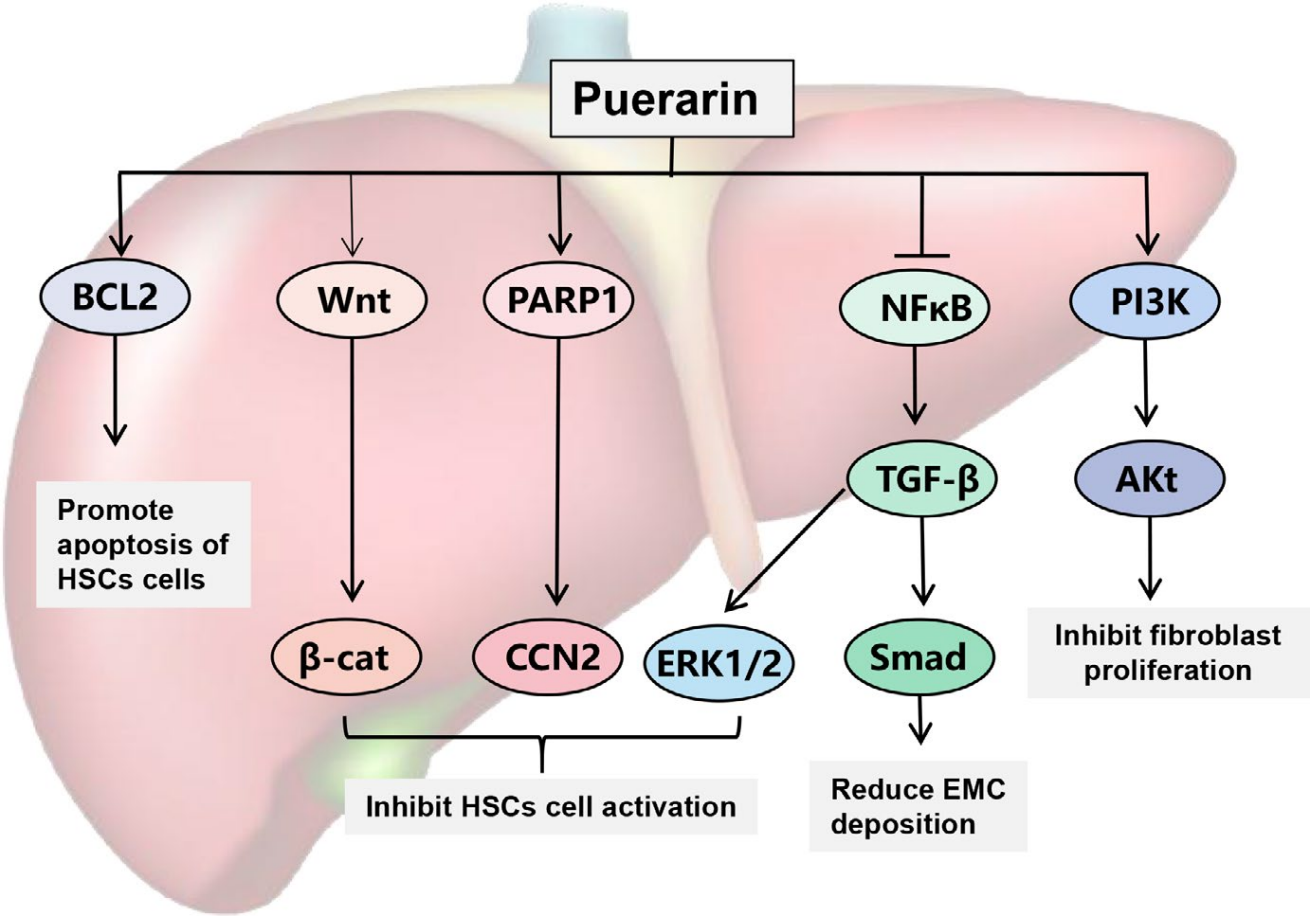
Molecular mechanism of puerarin against liver fibrosis. BCL‐2, B‐cell lymphoma‐2; PARP‐1, poly ADP‐ribose polymerase1; CCN2, cellular communication network factor 2; ERK1/2, extracellular regulated protein kinases; Smad, drosophila mothers against decapentaplegic protein; TGF‐β, transforming growth factor‐β; PI3K, phosphoinositide 3‐kinase; EMC, expressive extracellular matrix

A variety of chronic injuries can lead to liver fibrosis, including viral hepatitis, alcohol abuse, and unhealthy eating habits (Hernandez‐Gea and Friedman 2011). As expected, puerarin has the effect of alleviating liver fibrosis (Figure 5).

It has been shown that hepatic stellate cells, as the primary source of the extracellular matrix after liver injury, are the main targets of liver fibrosis (Higashi et al., 2017). Puerarin can promote the apoptosis of HSC by down‐regulating the expression of BCL‐2 mRNA, inhibit the expression of PARP‐1 and up‐regulate the expression of PPAR‐γ, and eventually reverse the CCl_4_‐induced liver fibrosis and inflammation (Guo et al., 2013; Wang et al., 2016; Zhang et al., 2006). Huang et al. also achieved remarkable results in the combined treatment of puerarin and vitamin D for CCl_4_‐induced liver fibrosis by inhibiting the activation of hepatic stellate cells through the Wnt1/β‐catenin pathway (Huang et al., 2018). Another study found that puerarin's regulatory effect on TGF‐β/Smad and ERK1/2 signaling pathway may also be one of the strategies for alleviating liver fibrosis, which brings hope for the treatment of liver fibrosis (Xiuqing Li et al., 2019; Xu et al., 2013).

##### Other protective effects of puerarin on liver

Based on glycyrrhetinic acid(GA)‐PEG‐PBLA, Xiao et al. prepared puerarin nanoparticles targeting the liver with GA receptors to mitigate hepatic ischemia–reperfusion injury by modulating the Toll4/NF‐κB signaling pathway (Xiao et al., 2018). Puerarin can activate autophagy by increasing LC3B‐II / I ratio and beclin‐1 protein level, thereby reducing the expression level of p62 protein and inhibiting LPS / D‐Gal‐induced apoptosis of mouse hepatocytes (Li et al., 2018). In addition, puerarin can also inhibit the signal transduction of mTOR and effectively reduce the apoptosis of hepatocytes in rats with experimental liver injury (Zhou et al., 2018).

#### 1.1.2 Daidzin and daidzein

Daidzin and daidzein belong to isoflavones, which are widely found in leguminous plants such as radix pueraria lobata and licorice. They have a variety of effects such as anti‐tumor, protection of cardiovascular and cerebrovascular, lowering blood lipid, inhibiting osteoporosis, inhibiting atherosclerosis, and regulating the endocrine (Sun et al., 2016). Daidzein is similar to human estrogen in chemical structure, so it is also called as phytoestrogen (Křížová et al., 2019). Due to the daidzin, the corresponding glucoside of daidzein, this paragraph focuses on the protective effect of daidzein on the liver.

##### Effects of daidzein on oxidative stress in liver

The antioxidant molecular mechanism of daidein is shown in Figure 3. A study investigated the effect of daidzein on the antioxidant defense system of 7,12‐dimethylbenz[a]‐anthracene (DMBA)‐induced oxidative stress in mice, which found that daidzein can play a liver protective role on DMBA‐induced oxidative stress through its antioxidant activity and reduction of apoptosis (Choi & Kim, 2009). Daidzein has a protective effect on D‐Gal‐induced oxidative damage in rat liver by improving the increase of D‐gal‐induced MAD‐protein adducts and the activity of SOD (Wong et al., 2007). Meanwhile, daidzein can also reduce the oxidative stress caused by cisplatin and has a protective effect on hepatotoxicity by reducing ALT, ADT, ALP, and MDA levels and increasing GSH, superoxide SOD, and CAT (Karale and Kamath 2017). In addition, other studies have found that daidzein may exert its antioxidant activity by activating transcription promoters to up‐regulate catalase and forming antioxidant metabolites 6‐oh‐daidzein and 3'oh‐daidzein, which generally exhibit higher antioxidant activity than parent compounds (Kampkötter et al., 2008; Rimbach et al., 2003; Röhrdanz et al., 2002).

##### Effects of daidzein on hepatic lipid metabolism

Liver X receptors (LXPs) are nuclear hormone receptors. It plays an important role in the process of lipid metabolism, cholesterol metabolism, glucose metabolism, and inflammatory response. Therefore, it is considered as a potential therapeutic target for liver diseases such as nonalcoholic fatty hepatitis, viral hepatitis, and liver fibrosis (He & Liu, 2013). The molecular mechanism of daidein regulating lipid metabolism is shown in Figure 4. There is a research demonstrated that daidzein can activate LXRs in mouse embryonic fibroblast and cause changes in cholesterol and lipid metabolism (Luo et al., 2018). In order to study the effect of daidzein on nonalcoholic fatty liver disease, mice were fed a high‐fat diet and supplemented with soybean flavone. The results showed that daidzein could decrease the liver lipid concentration and reduce the liver steatosis (Kim et al., 2011). The hepatic microarray showed that the daidzein prevented liver steatosis by down‐regulating the production of carbohydrate reactive element‐binding protein that determines the synthesis of newborn fats. In addition, daidzein can increase anti‐adipohepatitis leptin and adiponectin mRNA levels by up‐regulating the expression of fatty acid β‐oxidation and anti‐adipogenesis genes, which reduces the concentration of adipogenic tumor necrosis factor‐α and ghrelin, so as to inhibit obesity (Kim et al., 2011; Niwa et al., 2010). These findings suggested that daidzein may mitigate nonalcoholic fatty liver disease by directly mediating liver new fat formation and altering adipocyte metabolism. In the study to analyze the effect of daidzein on diet‐induced obesity, rats were fed a high‐fat diet and treated with daidzein for 14 days. The results showed that daidzein could reduce the weight and fat content of liver and affect the activity of lipase, especially the key enzyme of obesity, stearyl CoA desaturase 1(SCD1), suggesting that daidzein has a protective effect on liver steatosis (Crespillo et al., 2011).

##### Other

Daidzein can reduce the proliferation of human liver cancer SK‐HEP‐1 cells and effectively induce the apoptosis of liver cancer cells through the mitochondrial pathway. The mechanism is to increase the release of mitochondrial cytochrome c and the activation of APAF‐1, caspase9, and caspase‐3 through the BCL‐2 family in liver cancer cells (Park et al., 2013). Although there is no direct evidence that daidzein has a protective effect on alcoholic liver disease, it can inhibit the free‐choice of ethanol intake in Syrian hamsters without significantly affecting the intake of water or food in hamsters (Keung & Vallee, 1993). This result suggested that daidein has the potential to be a safe and effective therapeutic agent to inhibit alcohol abuse and prevent the occurrence of alcoholic liver disease.

#### 1.1.3 Formononetin

Formononetin is one of the active ingredients in Pueraria lobata, astragalus, and angelica. Fen Jin et al. (Jin et al., 2017) found that formononetin significantly reduced acetaminophen (APAP)‐induced liver cell damage and apoptosis in mice, and played an antioxidant role by stimulating the expression of Nrf2. Further studies showed that formononetin could reverse the decrease in cell viability and glutathione content of LO2 cells exposed to acetaminophen APAP. These results suggest that formononetin has a protective effect on APAP‐induced hepatotoxicity. Another study showed that, by reaction with NF‐κB/pAkt signaling molecules, formononetin can regulate oxidative stress, inflammatory response, cell apoptosis, reverse tissue degeneration, and attenuate Rit‐induced hepatotoxicity in *SD* rats, which indicates that it has a therapeutic role in hepatotoxicity and other hepatocyte diseases (Alauddin et al., 2018). ShuYang et al. (Shu Yang et al., 2019) found that formononetin can have a protective effect on anti‐induced liver injury by improving bile acid metabolism in the liver and the whole body. Its mechanism is to up‐regulate the expression of SIRT1 and activate PPARα to improve liver cholestasis. Formononetin can also significantly improve liver steatosis in mice a fed high‐fat diet. By activating AMPK, formononetin promotes nuclear translocation of key regulator transcription factor EB (TFEB) in lysosomal organisms, leading to autophagosome–lysosomal fusion and lipid phagocytosis, and alleviates hepatic steatosis (Yan Wang et al., 2019).

#### 1.1.4 Genistein

Genistein is an active ingredient extracted from Pueraria lobata, soybean, and other plants. It has a variety of physiological functions, which is widely used in pharmaceutical and healthcare products (Ganai & Farooqi, 2015).

Genistein can significantly reduce D‐Gal‐induced the chronic liver injury and liver fibrosis, and its mechanism may be to regulate the TGF/Smad signaling pathway. The result suggested that genistein can become a new therapeutic agent for this type of disease (Ganai & Husain, 2017). To determine whether genistein could reduce experimental liver fibrosis and cholestasis, Alfonso et al. ligated the common bile duct of rats and began administration of genistein 4 weeks after biliary obstruction. The results showed that genistein could inhibit hepatic fibrosis and cholestasis caused by prolonged bile duct obstruction in rats (Salas et al., 2007). Therefore, it has the potential to treat liver fibrosis. The deposition of schistosoma eggs in the liver leads to the formation of liver granuloma and fibrosis. Genistein can improve the liver granuloma and fibrosis of schistosomiasis by inhibiting the activity of NF‐κB signaling pathway, and it may be a potential natural drug to prevent this disease (Wan et al., 2017).

Genistein can protect APAP‐induced hepatotoxicity by activating the activities of UDP‐glucuronyl transferase (UGTs) and glutathione peroxidase and inhibiting CYP2E1, thereby inhibiting the biotransformation of APAP and the oxidative stress response of the liver(Fan et al., 2013). Morphine is one of the most potent painkillers in the world, which can cause liver damage through oxidative stress. Studies have shown that genistein, as an antioxidant and effective phytoestrogen, can resist the harmful effects of morphine on the liver of mice by improving enzyme activity and liver function (Salahshoor et al., 2018). The high rate of postoperative recurrence of hepatocellular carcinoma (HCC) is a major obstacle to the prognosis. Genistein can down‐regulate matrix metalloproteinase‐2 (MMP‐2), thereby reducing the proliferation and metastasis of hepatocellular carcinoma cells (Chen et al., 2013). In order to investigate the protective effect of genistein on chronic alcoholic liver injury, Liang Zhao et al. injected alcohol into the stomach of mice every day for 5 weeks and treated them with genistein. The results indicated that genistein effectively alleviated chronic alcoholic liver injury through its potential antioxidant, anti‐inflammatory, and anti‐apoptotic effects (Zhao, et al., 2016). Moreover, genistein has a protective effect on hepatic steatosis induced by high‐fat and high‐sucrose diets in rats. It can effectively regulate dyslipidemia and reduce fat accumulation in the liver by regulating AMPK and its related pathways (Liu et al., 2017).

#### 1.1.5 Soyasaponins

Soyasaponins are common saponins widely existing in legumes. According to the structure of saponins, it can be divided into A group, B group, E group, and DDMP group. Many studies have proved that soy saponins are a kind of natural biological active substance with comprehensive application value, which have the effects of immune enhancement, anti‐aging, antioxidation, and anti‐tumor effects (Isanga & Zhang, 2008; Singh et al., 2017; Xiang et al., 2006).

Soy saponins can enhance the activities of superoxide dismutase and glutathione peroxidase to reduce the production of ROS (Chen et al., 2014). Meanwhile, it can reduce the activity of ALT and AST in the serum of rats with precancerous lesions, increase the activity of SOD, CAT, and GSH‐px and reduce the content of MDA and NO in the serum (Hui‐ & Jin, 2012). Other studies have shown that soy saponins can alleviate liver injury induced by D‐Gal in mice, and the mechanism may also be related to diminishing YB‐1 phosphorylation and Nlrp3‐ inflammasome priming (Wang et al., 2020). In addition, soy saponins also have a protective effect on mice with acute alcoholic liver injury, which can reduce oxidative damage and inhibit the occurrence of steatosis and inflammation (Yang et al., 2011). In conclusion, these results indicate that soy saponins may inhibit the formation of precancerous lesions in rats through its antioxidant effect.

## 2 CONCLUSION

The liver is the largest substantive organ in the human body, and its functions are very complex, including metabolism, synthesis, biological transformation, secretion, and excretion of bile. As the first organ in the small intestine to come into contact with foreign substances after absorption, the liver is also a highly vulnerable organ. In recent years, with the improvement of people's living standards, changes in diet structure and living environment, the incidence of fatty liver, cirrhosis, liver cancer, and other liver diseases are also increasing. Many traditional Chinese medicines have the function of protecting the liver. As a multi‐component and multi‐target system, traditional Chinese medicine treatment has become an important strategy for the clinical treatment of a variety of chronic diseases, including liver disease.

Pueraria lobata is a widely used Chinese herbal in traditional Chinese medicine, with a long history been used in both food and medicine, which has the reputation of "Asian ginseng." It was recommended in the compendium of materia medica by Li Shizhen in 1596 as an anti‐alcohol drug. As a widely used Oriental traditional Chinese medicine, Pueraria lobata, its chemical composition has been widely studied. Pueraria lobata contains many natural active products, among which flavonoids are the most concerned. Flavonoids are widely found in a variety of plants and have a variety of pharmacological activities and low toxicity. It has remarkable effects to alleviate liver injury, induced by drugs, chemicals, and alcohol. It has become a hot topic in the treatment of liver injury in recent years. A large number of basic studies have shown that a variety of natural active products of Pueraria lobata have a certain protective effect on different types of liver diseases (Table. 1). The main active components of several Pueraria lobata discussed in this review, such as puerarin, daidzein, formononetin, genistein, and soy saponins, can play crucial roles in liver protection by regulating oxidative stress, inflammatory response, and lipid metabolism. However, the molecular mechanism and target of some pharmacological properties of these components are still unknown, which limits the further development of clinical application of pueraria lobata.

**TABLE 1:** Protective effect of isoflavones and triterpenoid saponins from pueraria lobata on liver diseases

Active ingredients	Model	Efficacy	Dose/Concentrations	Targets/Pathway/Mechanism	Reference
Puerarin	Alcoholic liver injury in rats	Reduces intestinal permeability and prevents alcohol absorption	3 g/kg/d (Crude extract of puerarin; purity, 58.9%)	Protects intestinal tight junction protein ZO−1	(Zhang et al., 2009)
Puerarin	Alcoholic liver injury in rats	Promotes alcohol metabolism	30, 60, 120 mg /kg/d	Increases the activity of ADH and ALDH; Reduces CYP2E1, CYP1A2, and CYP3A levels	(Chen et al., 2013)
Puerarin	Alcoholic liver injury in mice	Alleviates oxidative stress	200 mg/kg/d	Increases GSH, SOD, and GPX levels	(Zhao et al., 2010)
Puerarin	Alcoholic liver injury in rats	Regulates inflammatory response	30, 60, 120 mg /kg/d	Decreases IL−1 β, IL−6, PGE2, and TNF‐α levels; inhibits GSK−3β/NF‐κB pathway	(Li et al., 2013)
Puerarin	The rat hepatoma cell line H4IIE treated with ethanol	Restores hepatocyte autophagy; regulates lipid homeostasis	60, 120 μM	Activates AMPK/mTOR pathway	(Noh et al., 2011)
Puerarin	Pb‐induced liver injury in rats	Alleviates oxidative stress; antiapoptotic	400 mg/kg/d	Increases the activity of GPx, CAT, SOD, and GSH; decreases caspase−3 level	(Liu et al., 2012)
Puerarin	Ni‐induced liver injury in mice	Alleviates oxidative stress; reduces inflammation	400 mg/kg/d	Promotes the expression of MT protein; decreases TNF‐α, IL−6, PGE2, and COX−2 levels; inhibits TLR4/p38/CREB pathway	(Liu et al., 2016)
Puerarin	CCl_4_‐induced liver injury in mice	Alleviates oxidative stress; reduces lipid peroxidation	200, 400 mg/kg/d	Activates JNK/c‐jun/CYP7A1 pathway	(Ma et al., 2014)
Puerarin	Type 2 diabetes mellitus in mice	Alleviates oxidative stress; improves liver energy metabolism	650 mg/kg/d	Upregulates bcl−2 expression; increases the activity of SOD, GSH‐Px, Na(+)‐K(+)‐ATPase	(Shuo Yang et al., 2009)
Puerarin	Nonalcoholic fatty liver disease induced by a high‐fat diet in rats	Improves liver steatosis	400, 800 mg/kg/d	Activates JAK2/STAT3 pathway	(Zheng et al., 2009)
Puerarin	Nonalcoholic fatty liver disease induced by a high‐fat diet in rats	Improves liver steatosis	50, 100, 200 mg/kg/d	Upregulates hepatic PPAR‐γ and IR expression levels	(Zhao et al., 2016)
Puerarin	Steatohepatitis induced by a methionine‐ and choline‐deficient diet in mice	Regulates the early stages of lipid metabolism	900 mg/kg/d	Increases the expression of PPAR‐γ	(Yunliang Wang et al., 2014)
Puerarin	CCl_4_‐induced liver injury in rats	Reverses liver fibrosis	400, 800 mg/kg/d	Down‐regulates bcl−2 mRNA expression; induces apoptosis of activated HSC cells	(Zhang et al., 2006)
Puerarin	CCl_4_‐induced liver injury in mice	Reverses liver fibrosis; alleviates oxidative stress	100, 200 mg/kg/d	Inhibits the expression of PARP−1	(Wang et al., 2016)
Puerarin	CCl_4_‐induced liver injury in rats	Reverses liver fibrosis	200, 400, 800 mg/kg/d	Upregulates the PPAR‐γ expression; blocks the PI3K/Akt pathway	(Guo et al., 2013)
Puerarin	CCl_4_‐induced liver injury in rats	Reverses liver fibrosis	400 mg/kg/d	Inhibits Wnt1/β‐catenin pathway; inhibits the activation of hepatic stellate cells	(Huang et al., 2018)
Puerarin	Dimethylnitrosamine‐induced liver injury in rats	Reverses liver fibrosis	500 mg/kg/d	Inhibits TGF‐β/Smad pathway	(Xu et al., 2013)
Puerarin	Thioacetamide‐induced liver injury in rats	Reverses liver fibrosis; Reduces inflammation	200 mg/kg/d	Inhibits TGF‐β/ERK1/2 pathway	(Xiuqing Li et al., 2019)
Puerarin	LPS/D‐Gal‐induced liver injury in mice	Restores the autophagy; reduces inflammation	200 mg/kg/d	Increases the ratio of LC3B‐II/I and the protein level of Beclin−1; decreases the level of p62 protein expression	(Li et al., 2018)
Puerarin	2‐AAF/PH‐induced liver injury in mice	Reduces apoptosis	200 mg/kg/d	Inhibits mTOR pathway via inactivation of AKT	(Zhou et al., 2018)
Daidzein	7,12‐dimethylbenz[a]‐anthracene‐induced liver injury in mice	Alleviates oxidative stress; reduces apoptosis	5, 25 mg/kg/d	Increases SOD, CAT, GSH‐Px, and GR levels; Decreases caspase−3 level and increases Bcl−2 level	(Choi et al., 2009)
Daidzein	D‐Gal‐induced liver injury in rats	Alleviates oxidative stress	100 mg/kg/d	Increases SOD activity; decreases MDA level	(Wong et al., 2007)
Daidzein	Cisplatin‐induced liver injury in rats	Alleviates oxidative stress	20, 40 mg/kg/d	Increases SOD, GSH, and CAT activity; Decreases MDA level	(Karale et al., 2017)
Daidzein	The rat hepatoma cell line H4IIE treated with H2O2	Alleviates oxidative stress	200, 300 μM	Increases CAT and GPx levels	(Röhrdanz et al., 2002)
Daidzein/Genistein	Western‐style diet‐induced obesity in mice	Regulates lipid metabolism	160 mg/kg/d	Activates liver X receptor	(Luo et al., 2018)
Daidzein	Nonalcoholic fatty liver disease induced by a high‐fat diet in mice	Regulates lipid metabolism	100, 500 mg/kg/d	Promotes leptin and adiponectin mRNA expression; activates AMPK/PPARα pathway	(Kim et al., 2011)
Daidzein	Nonalcoholic fatty liver disease induced by a high‐fat diet in rats	Reduces steatosis	50 mg/kg/d	Promotes leptin and adiponectin mRNA expression; activates PPARα and PPARγ	(Crespillo et al., 2011)
Daidzein	Human hepatocellular carcinoma cells SK‐HEP−1	Induces the apoptosis	200, 400, 600 μM	Increases the release of mitochondrial cytochrome c and the activation of APAF−1, caspase9, and caspase−3 through the bcl−2 family	(Park et al., 2013)
Formononetin	APAP‐induced liver injury in mice	Alleviates oxidative stress	50, 100 mg/kg/d	Increases GSH activity; activates Nrf2 pathway	(Jin et al., 2017)
Formononetin	Ritonavir‐induced liver injury in rats	Modulates the oxidative stress, inflammation, apoptosis and reversing the tissue degeneration	100 mg/kg/d	Attenuates the RIT‐induced Bax, caspase−3, NFκB, and eNOS activation; upregulates the Bcl−2 and pAkt level	(Alauddin et al., 2018)
Formononetin	Naphthalene isothiocyanate‐induced liver injury in mice	Ameliorates cholestasis; reduces inflammation	10, 20, 50 mg/kg/d	Activates SIRT1; regulates PPARα/JNK pathway	(Shu Yang et al., 2019)
Formononetin	Nonalcoholic fatty liver disease induced by a high‐fat diet in mice	Regulates lipid metabolism; promotes autophagy	100 mg/kg/d	Activates AMPK/TFEB pathway; facilitates TFEB‐mediated lysosome biogenesis	(Yan Wang et al., 2019)
Genistein	D‐GalN‐induced liver injury in rats	Improves liver fibrosis	5 mg/kg/d	Modulates the expression of Smad2/3 and Smad7; activates Smad/TGF‐β pathway	(Ganai et al., 2017)
Genistein	Liver injury by prolonged biliary obstruction in rats	Decreases liver fibrosis and cholestasis	25 mg/kg/d	Increases Matrigel and collagen type I degradation	(Salas et al., 2007)
Genistein	Schistosomiasis‐induced liver granuloma and fibrosis in mice	Reduces liver granuloma and fibrosis	25, 50 mg/kg/d	Decreases MCP−1, TNFα, IL1β, IL4, CXCL1 and IL10 mRNA levels; inhibits NF‐κB pathway	(Wan et al., 2017)
Genistein	APAP‐induced liver injury in mice	Alleviates oxidative stress	50, 100, 200 mg/kg/d	Increases the activities of GSH, UGTs, GSH‐PX, and TAC; inhibits CYP2E1; activates UDP‐glucuronosyltransferase; accelerates the glucuronidation of APAP	(Fan et al., 2013)
Genistein	Morphine‐induced liver injury in mice	Reduces liver damage	25, 50 mg/kg/d	Reduces serum AST, ALT, ALP, and NO levels	(Salahshoor et al., 2018)
Genistein/Puerarin	Alcoholic liver injury in mice	Alleviates oxidative stress; inhibits lipid peroxidation; reduces apoptosis	0.3 mmol/kg/d	Increases HO−1, CAT, SOD, GSH, and GSH‐Px levels; decreases NF‐κB p65, TGF‐β1, COX−2, MCP−1, TNF‐α, and IL−6 levels	(Zhao et al., 2016)
Genistein	Nonalcoholic fatty liver disease induced by a high‐fat diet in rats	Improves liver steatosis	4, 8 mg/kg/d	Activates AMPK/PPARα pathway; decreases the expression of SREBP−1c	(Liu et al., 2017)
Soyasaponins	BRL cells treated with H_2_O_2_	Alleviates oxidative stress	25–800 μg/mL	Increases SOD, GSH‐Px, GSH, and CAT levels; reduces ROS	(Chen et al., 2014)
Soyasaponins	precancerous lesion of liver in rats	Increases antioxidant activity	100 mg/kg/d	Increases CAT, SOD, and GSH‐Px levels; Decreases MDA and NO levels	(Hui‐ et al., 2012)
Soyasaponins	D‐GalN‐induced liver injury in mice	Reduces inflammation and immune response	5 mg/kg/d	Diminishes YB−1 phosphorylation and Nlrp3‐ inflammasome priming	(Wang et al., 2020)
Soyasaponins	Alcoholic liver injury in mice	Alleviates oxidative stress; improves liver steatosis	300, 600, 1200 mg/kg/d	Reduces TC, TG, and MDA levels; increases SOD, GSH‐Px, and GSH levels	(Yang et al., 2011)

To sum up, although the current research on Pueraria lobata is still insufficient, we positively believe that its natural products still have broad application prospects in the treatment or prevention of liver disease. However, in order to further promote the application and development of Pueraria lobata, we still need to conduct more in‐depth research on its liver protection mechanism.

## CONFLICT OF INTEREST

The authors declare that they have no competing financial interests or personal relationships that could have appeared to influence the work reported in this paper.

## AUTHOR CONTRIBUTION

**Heng He:** Writing‐original draft (lead). **Shuwei Peng:** Writing‐review & editing (equal). **Xu Song:** Investigation (equal); Methodology (equal). **Renyong Jia:** Resources (equal). **Yuanfeng Zou:** Supervision (equal). **Lixia Li:** Supervision (equal). **Zhongqiong Yin:** Project administration (equal).

## ETHICAL APPROVAL

Not applicable.

## Data Availability

Data sharing is not applicable to this article as no new data were created or analyzed in this study.
